# Macroscopic Pulmonary Fat Embolism Secondary to Intraosseous Line Placement: A Case Report

**DOI:** 10.1155/crcc/7302079

**Published:** 2026-06-08

**Authors:** Stephanie J. Chen, Andrew C. Sanchez, Serena T. Hua, Joel N. Murala, Charisma J. Kaushik, Amit Banga, Gundeep S. Dhillon

**Affiliations:** ^1^ Division of Pulmonary, Allergy and Critical Care Medicine, Stanford University, Stanford, California, USA, stanford.edu; ^2^ Department of Pathology, Stanford University, Stanford, California, USA, stanford.edu; ^3^ Divison of Emergency Medicine, The Permanente Medical Group, Inc., Oakland, California, USA, kaiserpermanente.org; ^4^ Department of Pharmacology and Physiology, Georgetown University, Washington DC, USA, georgetown.edu; ^5^ Department of Critical Care Medicine, Stanford University, Stanford, California, USA, stanford.edu

## Abstract

**Background:**

Intraosseous access is commonly used for vascular access in emergent settings. It is generally thought to be well tolerated with minimal complications. We report the first case of clinically significant macroscopic pulmonary fat embolism secondary to intraosseous access.

**Case Presentation:**

A 67‐year‐old woman developed profound hypotension and severe biventricular dysfunction shortly after intraosseous access and resuscitation. She required emergent venoarterial extracorporeal membrane oxygenation and Impella for cardiopulmonary support. Computed tomography revealed a new macroscopic pulmonary fat embolism compared with prior imaging 1 day prior. Aspiration thrombectomy was successfully performed with significant improvement in hemodynamics. Unfortunately, she suffered an anoxic brain injury during resuscitation and was ultimately transitioned to comfort care.

**Conclusions:**

Although intraosseous access is often considered a safe procedure, this case highlights the need for awareness of this rare but serious and potentially lethal complication. Treatment of pulmonary fat embolism is often supportive; however, aspiration thrombectomy has a potential therapeutic role in macroscopic cases.

## 1. Introduction

Intraosseous (IO) access is a potentially life‐saving vascular access modality often used in trauma or advanced cardiopulmonary life support situations when rapid intravenous access is needed but standard intravenous access cannot be secured [1]. It is generally well tolerated, with minimal complications, mainly pain or extravasation. Fat embolism syndrome (FES) is uncommon but lethal, often associated with long bone fractures [2]. Most cases of FES are microscopic, with rare case reports describing macroscopic fat embolism after cosmetic procedures and long bone fractures [3]. We present the first case of macroscopic pulmonary fat embolism in an adult patient secondary to IO line insertion during cardiopulmonary resuscitation. This case was previously presented as a preprint [4].

## 2. Case Presentation

A 67‐year‐old woman, with a body mass index (BMI) of 26, who had undergone bilateral lung transplant for pulmonary fibrosis 2 months prior and remained hospitalized during her posttransplant course, was recovering well and awaiting discharge. During the week of discharge planning, she developed acute fevers and persistent hypotension. Fluid resuscitation was limited given the setting of chronic pleural effusions, and she was transferred to the medical intensive care unit where she was started on low‐dose norepinephrine and antibiotics. Two days after being transferred to the ICU, she was noted to be more somnolent with new respiratory acidosis (pH 7.18, pCO2 74.8) and was placed on bilevel positive pressure ventilation. Five hours later, she developed rapidly worsening hypotension requiring high dose norepinephrine (0.1 mcg/kg/min) and vasopressin drips (0.06 units/min). She remained somnolent, so the decision was made to proceed with endotracheal intubation. Her intubation was complicated by epistaxis and difficulty with bag ventilation due to blood clots in the endotracheal tube, resulting in bradycardia with heart rate of 30 s and subsequent cardiac arrest. Chest compressions were initiated. At this time, an Arrow EZ‐IO power driver (Teleflex) was used to place a 45‐mm, 15G IO needle in the left proximal tibia given limited vascular access and ongoing active compressions. IO placement was prioritized over central venous catheterization, as it was felt to offer a faster and easier route for resuscitative medication delivery. Insertion was performed using standard technique with advancement until loss of resistance was felt upon entering the medullary cavity. Placement was successful after one attempt with aspiration of marrow. The line flushed easily without evidence of extravasation. A 1 liter bolus of crystalloid fluid was administered through the IO line under pressure. Return of spontaneous circulation was achieved after two rounds of cardiopulmonary resuscitation. However, she remained persistently hypotensive on norepinephrine (0.2 mcg/kg/min) and vasopressin (0.06 units/min) drips. The post intubation X‐ray revealed a new large right basilar hydropneumothorax, and after a pigtail chest tube was placed, her hemodynamics significantly improved, and she was able to be weaned off norepinephrine and vasopressin drips.

About 1 hour later, she developed profound hypotension requiring multiple pushes of 1 mg of epinephrine as well as resumption of high‐dose norepinephrine (0.3 mcg/kg/min) and vasopressin (0.04 units/min) drips. She was found to have a new severe biventricular dysfunction on transesophageal echocardiogram and epinephrine drip was started, up to 0.25 mcg/kg/min. She was emergently placed on venoarterial extracorporeal membrane oxygenation (VA‐ECMO) and Impella for further cardiopulmonary support. A whole‐body computed tomography (CT) scan revealed a new left femoral to external iliac nonocclusive deep venous thrombosis (DVT) and multiple filling defects in the right lower lobar pulmonary artery and bilateral upper segmental pulmonary arteries with attenuation of fat (approximate Hounsfield units −86; approximate Hounsfield units for fat: −100 to −60 vs. soft tissue/blood: +25 to +60), consistent with macroscopic pulmonary fat embolism (Figure 1). Neither finding was present on whole‐body CT scan the day prior.

**Figure 1:**
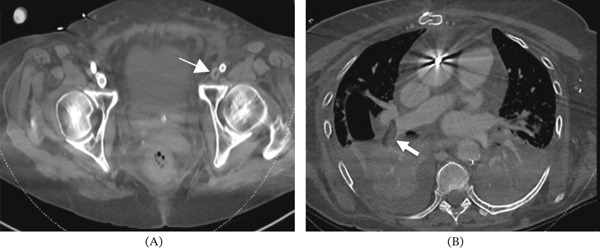
(A) Pelvic computed tomography coronal images with arrow pointing to new nonocclusive left femoral/external iliac deep vein thrombosis after IO insertion. (B) Computed tomography of chest coronal images with arrow pointing to large right lower lobe pulmonary artery embolus with fat attenuation (mean Hounsfield units −81.67), consistent with macroscopic pulmonary fat embolus.

The patient underwent aspiration thrombectomy of the right lower lobe pulmonary artery by interventional radiology with removal of rubbery/malleable material measuring 3.8 × 2.5 × 0.6 cm in aggregate. Pathology revealed fat admixed with clot, consistent with a fat embolus (Figure 2). After aspiration thrombectomy, her hemodynamics improved significantly and she was successfully weaned off ECMO, Impella, vasopressors, and inotropes. Unfortunately, she suffered anoxic brain injury during resuscitation and was ultimately transitioned to comfort care.

**Figure 2:**
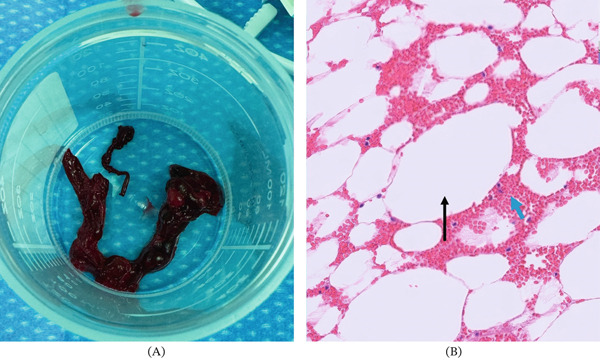
(A) Gross specimen of aspirated material from right lower lobe. (B) Hematoxylin and eosin staining of aspirate material at 200× magnification showing fat admixed with blood clot. The black arrow indicates an adipocyte, and the blue arrow indicates red blood cells.

## 3. Discussion

FES is an uncommon but potentially lethal phenomenon that is characterized by the classic triad of hypoxemia, neurologic abnormalities, and petechiae [2]. It develops after an insult leads to the release of fat into the circulation. FES is commonly associated with orthopedic traumas, typically long bone leg fractures, with an estimated incidence ranging from 0.54–1.29% depending on location and number of fractures. Most cases of fat embolism are microscopic, with CT chest imaging findings of smooth septal thickening, geographic crazy‐paving areas, and lack of filling defects in the pulmonary arteries. Macroscopic fat embolism with fat attenuation filling defects on CT pulmonary angiography is rare, with few case reports describing findings after cosmetic procedures and long bone fractures [3, 5]. While rib fractures have also been associated with fat pulmonary embolism, this patient only had chronic rib fracture deformities poststernotomy, and no acute rib fractures on CT after chest compressions were performed. Unfortunately, there was no imaging of her lower leg to assess for possible new long bone fracture associated with IO placement. Other recognized nontraumatic causes of fat embolism, including sickle cell disease and parenteral lipid infusion, were also absent [2, 6]. Since the left‐sided DVT was new from the day prior and the aspirated pathology showed fat admixed with clot, we hypothesize that IO placement and disruption of the natural bone architecture provided a conduit for marrow fat to be introduced into the vasculature, especially as rapid pressurized infusions increase intramedullary pressure leading to further microscopic fractures and microvascular disruption between marrow fat and sinusoids [6].

Several patient‐specific risk factors may have further predisposed our patient to development of fat embolism. In pediatric literature, elevated BMI has been associated with increased risk of fat embolism [7]. Although her BMI classified her as only mildly overweight, she had been on corticosteroids for several months following transplantation and had significant muscle loss with relatively increased adiposity. Additionally, given her prolonged hospitalization, she had significant deconditioning and muscle loss, further increasing the risk for bone fragility. Chest tube placement or endotracheal intubation does not disturb bony structure and has not been reported to have an association with fat embolism.

IO access is widely used in resuscitation and considered relatively safe, with prior studies reporting < 1% rate of serious complications. Commonly reported complications include patient discomfort/pain, bent/broken needle, and fluid extravasation. Serious complications include long bone fracture, compartment syndrome, and osteomyelitis [8]; however, a recent systematic review demonstrated that pulmonary fat emboli after IO insertion were common, with 83% incidence across seven animal studies [9]. Clinical relevance in humans remains unclear. One pediatric autopsy study found greater than 50% incidence of pulmonary fat embolism associated with IO insertion during resuscitation [6]. The significance of this finding is difficult to interpret, given the inherent challenges in attributing cause of death during resuscitation. It remains unclear whether these fat embolisms contributed to mortality or were incidental findings from the IO placement during resuscitation.

This case demonstrates a severe complication of IO access that has not previously been described in the human adult population and should serve as a sentinel event. Given the severity and potential lethality of IO access described in this case, careful consideration should be given before IO placement, with attention to depth of insertion to not penetrate the posterior cortex. While a helpful modality especially in out‐of‐hospital cardiac arrests or clinical scenarios with few code team members, at centers where code teams have abundant personnel, it may be important to consider deferring IO placement if other venous access can be achieved within 5 minutes or to consider intramuscular administration of medications temporarily, so long as high quality compressions are ongoing. Moreover, if an IO is placed, it may be prudent to avoid pressure infusions as possible and stop use once other intravenous access is achieved to reduce the likelihood of clinically significant fat embolism.

In cases of traditional microscopic fat embolism leading to FES, treatment is solely supportive care, though aspiration thrombectomy can be a potential definitive treatment option in macroscopic pulmonary fat embolism. In the setting of cardiopulmonary collapse, extracorporeal membrane oxygenation can be utilized as a bridge while awaiting more definitive therapies.

In conclusion, we present the first reported case of a clinically significant macroscopic pulmonary embolism secondary to IO access in an adult human. Although IO access is generally regarded as a safe procedure, it is important to have a high degree of suspicion for this rare and potentially lethal complication.

**Nomenclature**
CT: computed tomography
DVT: deep vein thrombosis
FES: fat embolism syndrome
IO: intraosseous
VA‐ECMO: venoarterial extracorporeal membrane oxygenation

## Author Contributions

All authors contributed substantially to the conception, drafting, and revision of this case report. S.J.C., A.C.S., S.T.H., C.J.K., A.B., and G.S.D. were involved in the clinical care of the patient. S.J.C. was involved in initial manuscript drafting. J.N.M. assisted with the literature review. All authors contributed to critical revision and approval of the final manuscript.

## Funding

No funding was received for this manuscript.

## Disclosure

A preprint of the case report has previously been published (Chen et al. 2025). The case report has never been published in a journal. The preprint of the case report is listed in the references [4].

## Consent

Written consent from family has been obtained and filed for the publication of this case report. The authors attest that their institution does not require Institutional Review Board approval for the publication of this case report.

## Conflicts of Interest

The authors declare no conflicts of interest.

## Data Availability

Data sharing is not applicable to this article as no datasets were generated or analyzed during the current study.
